# Risk adjustment and observation time: comparison between cross-sectional and 2-year panel data from the Medical Expenditure Panel Survey (MEPS)

**DOI:** 10.1186/2047-2501-2-5

**Published:** 2014-07-25

**Authors:** Yi-Sheng Chao, Chao-Jung Wu, Tai-Shen Chen

**Affiliations:** School of Public Health, University of Alberta, Edmonton, Alberta T6G2T4 Canada; Department of Cell Biology, University of Alberta, Edmonton, Alberta T6G2T4 Canada; Division of Biometry, Department of Agronomy, National Taiwan University, Taipei, Taiwan

## Abstract

**Background:**

Risk adjustment models were used to estimate health care consumption after adjusting for individual characteristics or other factors. The results of this technique were not satisfying. One reason could be that the length of time to document consumption might be associated with the mean and variance of observed health care consumption. This study aims to use a simplified mathematical model and real-world data to explore the relationship of observation time (one or two years) and predictability.

**Methods:**

This study used cross-sectional (one-year) and 2-year panel data sets of the Medical Expenditure Panel Survey (MEPS) from 1996 to 2008. Comparisons of the health care consumption (total health expenditure, emergency room (ER) and office-based visits) included ratios of means and standard errors (SEs). Risk adjustment models for one- and two-year data used generalized linear model.

**Results:**

The ratios of mean health care consumption (two-year to one-year total expenditure, ER and office-based visits) seemed to be two in most age groups and the ratios of SEs varied around or above two. The R-squared of two-year models seemed to be slightly better than that of one-year models.

**Conclusions:**

We find health expenditure and ER or office-based visits observed in two consecutive years were about twice those observed in a single year for most age, similar to the ratios predicted in mathematical examples. The ratios of mean spending and visits varied across age groups. The other finding is that the predictability of two-year consumption seems better than that of one-year slightly. The reason is not clear and we will continue studying this phenomenon.

**Electronic supplementary material:**

The online version of this article (doi:10.1186/2047-2501-2-5) contains supplementary material, which is available to authorized users.

## Introduction

### Risk adjustment

According to Van de Ven and Ellis, risk adjustment was defined as “the use of information to calculate the expected health expenditures of individual consumers over a fixed interval of time” [1]. This is a technique using available individual characteristics to estimate propensities of consuming health care, such as expenditure and visits. With proper use, the potential benefit of risk adjustment include reducing selection by insurers (risk selection) and health plan seekers (adverse selection) [2, 3]. In a competitive market with perfect information, risk adjustment is a tool to achieve efficiency and fairness through providing adequate risk-adjusted premiums and cross subsidies between risk groups [1]. The other benefits include supporting financial stability of the system and providing consumer choices [4].

Currently risk adjustment mechanisms adopt different sets of independent variables to estimate health care consumption [1]. Basic models use demographic information, such as the Swiss system using gender and age to assess risks of different groups [5]. More complicated system requires other information. For example, the German system uses age, gender, disability, and entitlement of sickness allowances [6]. In the United States, there are diagnosis-based models to better adjust risks [7, 8]. Other models may use health expenditure in the previous year to make more precise predictions [1]. The best predictability (in terms of R-squared) achieved could be higher than 0.30 with diagnostic group prediction models. However, the performance of risk adjustment models remained unsatisfying [1, 9].

There are many reasons why risk adjustment could not perform as well as we expected [9]. One of the reasons was because the length of time might not be properly taken into account. The probability of observing a health event happening in life is first determined by the likelihood of this event to occur and then captured by timing of observation. For the first factor (disease development in life), the evidence from life course epidemiology showed that risk of certain events increased in the latter life long after some childhood or neonatal conditions [10]. A typical example is chronic conditions that fit a sensitive period model and are more easily to develop in a certain period in life [11]. This showed conditions may develop with some degree of certainty, but may not be fully predicable. The second factor is timing of observation. Evidence indicated that the length of time to capture health spending might itself influence the precision of the expenditures incurred. For example, the partial year coverage, monthly, has a higher monthly variance than those with a full year of information [2]. Currently most studies and insurance policies hold one assumption: health spending can be analyzed on a yearly basis and captured without causing any bias related to the length of observation time [9]. In the next section, this study hypothesized a scenario to illustrate the uncertainty caused by the length of time.

### Observational bias due to lengths of observation time

For risk adjustment, the length of observation time can induce an observation bias due to the uncertainty of observing a health condition cause health care consumption in life course. Assuming a health event (denoted as event *a*) is certain to develop in a multi-year (*n*) period of life course in a population, this event can incur a hypothesized and normally distributed amount of health expenditure [ ]. The probability of the insurers or health care providers to record the amount incurred by individuals with this event in any given 12-month period are assumed to be normally distributed,  in this case. Similarly, the probability of the third party to record the amount incurred by individuals with this event in a two-year period (including the 12-month period mentioned above) are assumed to be normally distributed, . The variance of *P(a*_*1*_*)* or *P(a*_*2*_*)* is assumed to be small enough to make the probability of being greater than one or less than zero unlikely.

In this hypothetical case, the insurers were likely to observe a product of health expenditure because of this event and probability of capturing it. After combining these two hypothesized normal variables [*Exp*(*Observed*_1*year*_) = *Exp*(*a*) × *P*(*a*_1_)], the amount of health expenditure incurred due to this condition in 12 month period should be the product of these two variables, assuming that expenditure and the probability of the insurers or health care providers to record expenditures are independent of each other. Compared to the observed two-year amount, *Exp*(*Observed*_2*years*_), the ratios of mean values and variance were as follows.

Ratios of mean values,
1

Ratios of variances,
2

The ratios of means are likely to be two, because the probabilities are proportional. The ratios of variances will be likely to be greater than one, because not only observation probability of 2 years (2/*n*) greater than that of one year (1/*n*), but also the 2-year observation may be associated with wider variability (for example, difficulty in recalling or documenting events more than one year ago).

The possible ranges of variance ratios depend on the expected mean values of health expenditure within *n* years (*μ*_*f*_), variances of probabilities (  and ). If the variances of observation probabilities within 12 months and 2 years were very similar for reasons like similar data collection precision and medical practice, the ratios of variances can be close to 1 with large *n* (true length of expenditure distribution).

In a more simplistic example assuming the amount of expenditure observed in one year is a constant and exactly half of the health spending incurred in two years (*μ*_*f*_*μ*_1_ = 0.5(*μ*_*f*_*μ*_2_)) without any observation bias or uncertainty ( ), the mean values of expenditures in two-year period are exactly twice as much as the spending distribution in 12-month period. The ratios of means and variances should be exactly two and four respectively. Compared to the hypothesized example, the result of shorter observation time (12 months versus 2 years) may lead to an observed distribution with disproportionally small variances (ratios of variances close to one, rather than four).

In sum, the probability of observing multi-year health events in a population possibly lead to disproportionally smaller variances and less predictability relative to those in two-year periods. However, how this observation bias due to the length of observation time might actually influence the spending distribution in real world was not verified and should be investigated. This study aims to 1) find out the variance change of spending distributions observed from 12-month to two-year periods; 2) quantify the predictability relationship between observed 12-month periods and 2-year panels; and 3) provide recommendations for risk adjustment models based on these findings. To test the hypothesis of observational bias, emergency room visits (assumed to be happen in single years, not multi-year events) and office-based visits (the most predictable measure of health care consumption [1, 4]) were also analyzed to compare the changes in mean values, variances and R-squared comparisons.

## Methods

### Model specification

Health expenditure distribution usually displays considerable skewness and the presence of observations containing zero or little expenditure [12]. It was concluded that the one-part GLM (log link) should be tried first based on considerations of efficiency and fit, as the two-part OLS models might have imprecise estimates due to a data transformation that was designed to adjust for heteroscedasticity and its variance structure [12]. Moreover, it was recommend to model health expenditure based on gamma distribution to better fit expenditure data [12]. ER and office-based visits were modeled with Poisson distribution.

### Functional forms

Based on the above discussion, this part of this analysis estimates conditional expenditure models. A general model specification could be expressed as follows:


The values of individual health expenditures among those with any consumption (*y*_it_) are estimated based on the vector of individual characteristics [*X*_*i*_] and time fixed effects (*T*) to capture any time-specific trends in Medicare spending. Time was defined as the year, from 1996 to 2008, when the data was collected (1-year data) or first collected (2-year panels).

Individual characteristics are defined differently in various studies. The risk adjusters based on the average annual per-capita cost (AAPCC) includes region^a^, gender, races, and age to get an average value of health spending in the region where individuals resided [12]. In other studies, the individual characteristics include gender, education, race, marital status, health status, [13] activities of daily living (ADL), instrumental activities of daily living (IADL), chronic conditions (stroke, heart disease, diabetes and others), [12] poverty status, [14] and residential characteristics [15] are used.

To make the results more comparable to these mentioned studies, there are two risk adjustment models. First, risks of all age groups (0 to 85 years) were adjusted with basic demographic information (gender, race and regions of residence). The other model adopted the above-mentioned variables for population groups age 45 to 65 years.

#### Model summary with R-squared

The other focus of this study is estimating the predictability change from one- to two-year observation lengths. However, the survey design in the MEPS data sets did not support direct estimation of R-squared. One solution to estimate predictability was to regress the actual spending with the predicted expenditure [16]. The observed amounts can be regressed with the predicted amounts from models to generate R-squared and to indirectly assess how much of the variation in the actual spending could be explained by the predicted values.

### Data

This study uses annual and 2-year panel (longitudinal) expenditure data from the Medical Expenditure Panel Survey (MEPS) that provides annual survey data from 1996 and follows up selected participants for 2 years to generate panel data [17]. The advantages of using MEPS annual and 2-year panel data sets include comparable data quality, national representativeness and specialization in health events and expenditure. However, the use of MEPS data sets requires the adjustment of survey design and careful choices of weight files.

### Data management

#### Data linkage of annual data sets

With the officially released linkage file (*h036b09*), it took several steps to integrate these annual files into a multi-year collection of individual spending. First, the relevant variables were selected from annual household component (HC) files of different years (from 1996 to 2008) [18]. The study period included thirteen years to match the number of panels available upon study. Then, these variables were assigned consistently with new variable names. Because most variables were named with year-specific labels, the observations in each year were preserved without year-specific tags thereby permitting empirical analyses. Second, the pooled observations from data sets were appended to each other. The final step was to match renewed sampling units and strata from the linkage file containing longitudinal weight. Merging with the linkage file introduced new structures of sampling units and strata to each participant that better address historical changes in US population.

#### Data linkage of 2-year panel data sets

First- and second-year data sets (HC files) of Panel One (data collected in 1996 and 1997 continuously) to eight (data collected in 2003 and 2004) were merged separately according to instructions [19] and data sets of Panel nine (data collected in 2004 and 2005) to thirteen (2008 and 2009) were available online [20]. Then, data sets of Panel one to thirteen were appended to one another to generate one single data set.

All statistical analyses were conducted with STATA 12 (STATA Corp, College Station, Texas).

## Results

**Figure 1:**
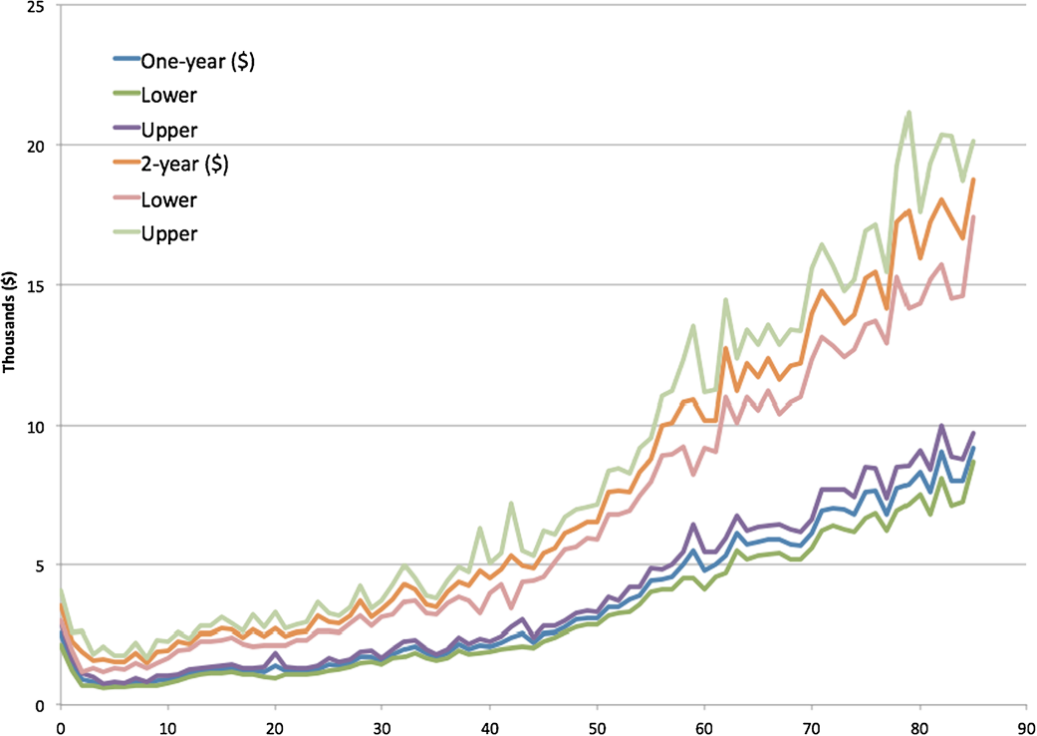
**Average health expenditure and the 95% confidence intervals observed in 1- and 2-year periods by age (0 to 85 years).**

Figure 1 (and Additional file 1: Table S1) presents the mean health spending of all ages (0 to 85 years) observed in one or two-year periods from 1996 (13 years or 13 2-year panels in total). The spending distribution looks like J-shape. Mean expenditure is high at age zero and then decreases with ages, until 10 years of age. The increase of health spending presents in both one- and two-year distribution. The standard errors of all ages seem to increase with the levels of spending and become larger at older ages.

**Figure 2:**
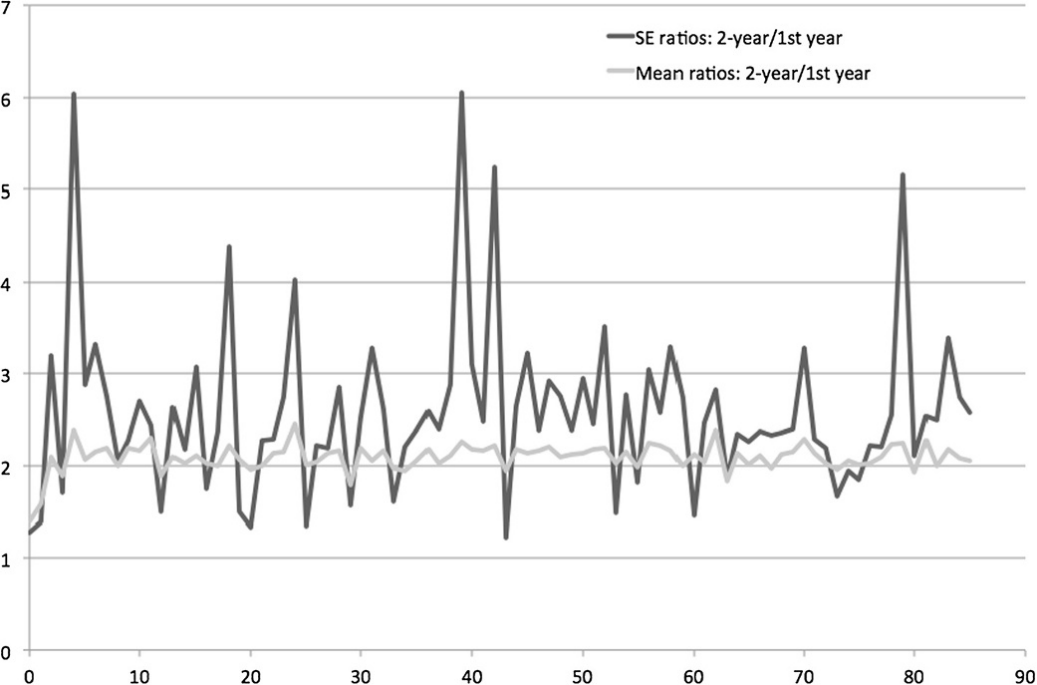
**Ratios of 1-year and 2-year mean health expenditure and standard errors by ages (0 to 85 years).**

**Figure 3:**
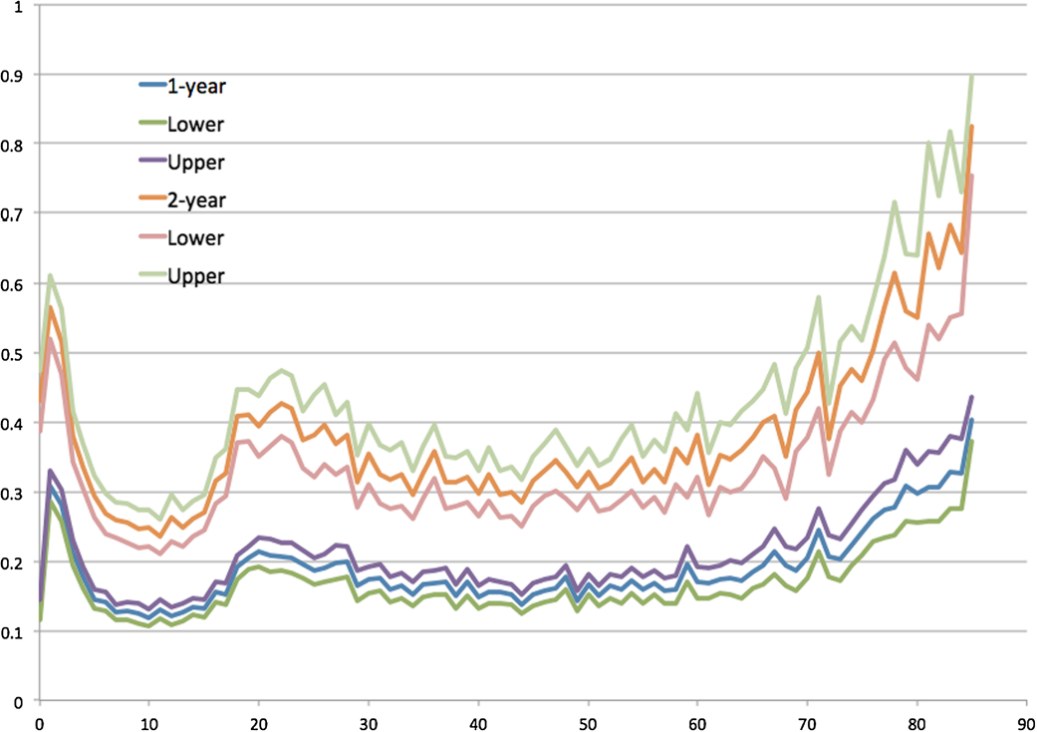
**Average emergency room visits and the 95% confidence intervals observed in 1- and 2-year periods by age (0 to 85 years).**

**Figure 4:**
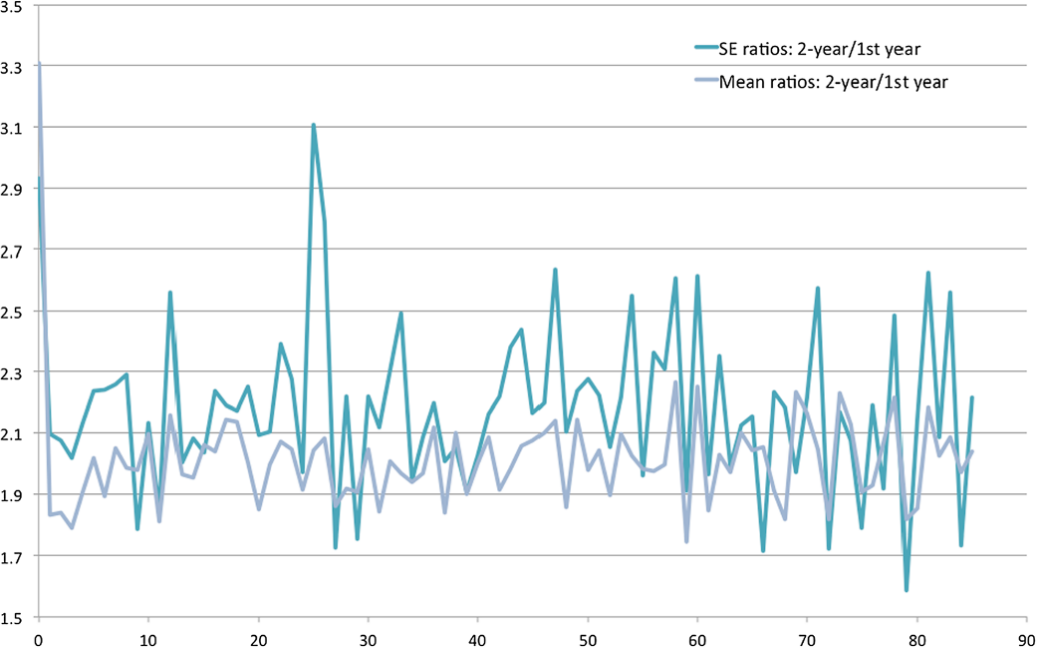
**Ratios of 1-year and 2-year mean emergency room visits and standard errors by ages (0 to 85 years).**

**Figure 5:**
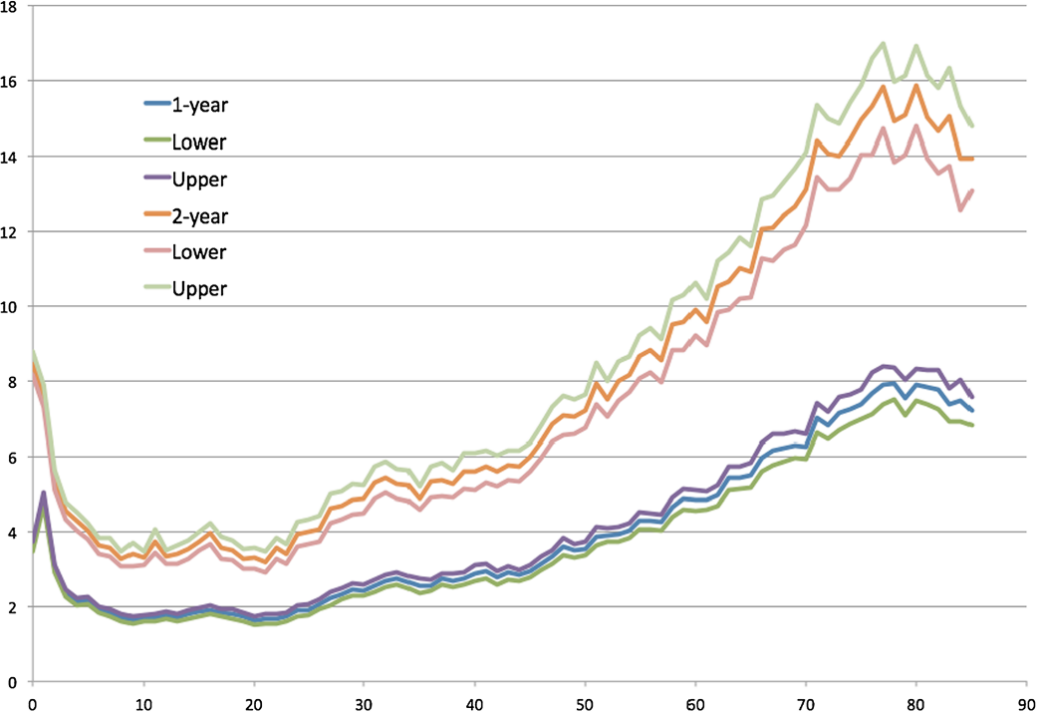
**Average office-based visits and the 95% confidence intervals observed in 1 and 2 years by age (0 to 85 years).**

**Figure 6:**
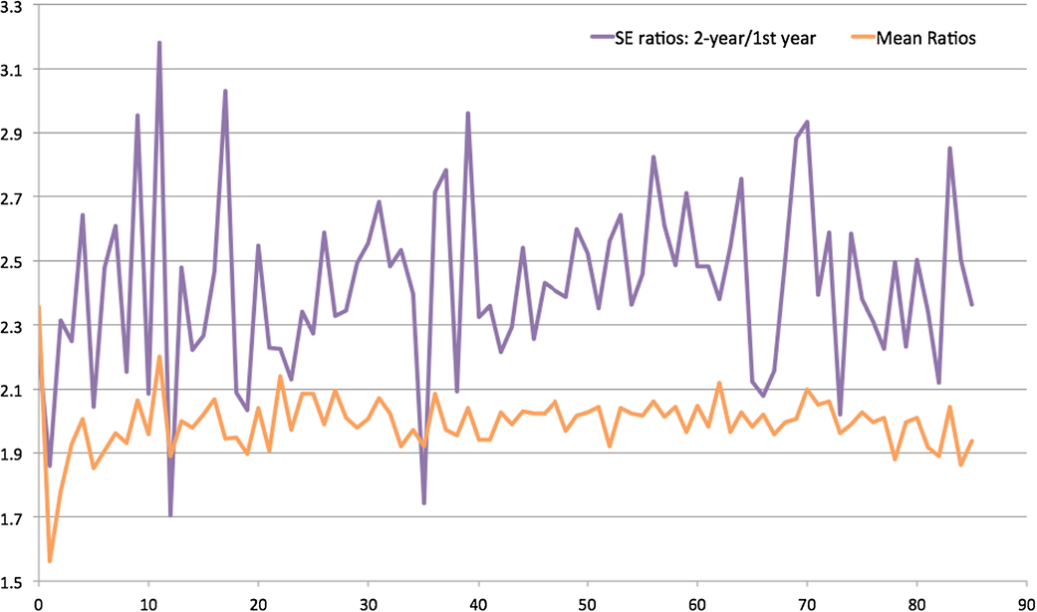
**Ratios of 1-year and 2-year mean office-based visits and standard errors by ages (0 to 85 years).**

**Figure 7:**
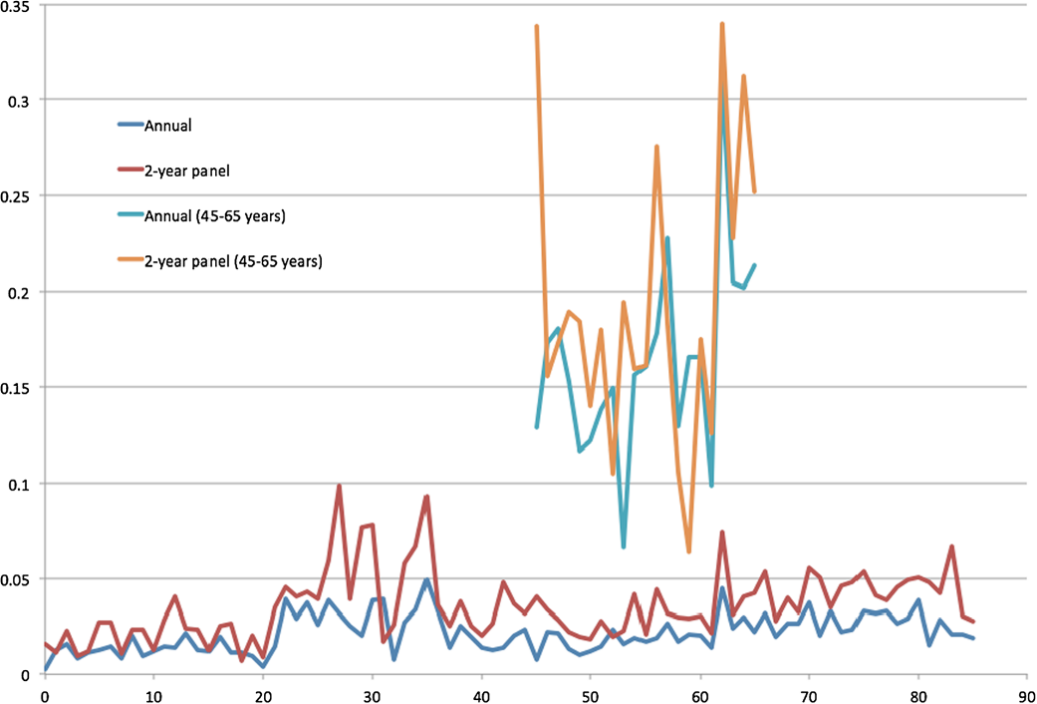
**R-squared estimated from 1- and 2-year health expenditure prediction by basic demographic characteristics for all ages and by basic and other variables for age 45 to 65 years.**

**Figure 8:**
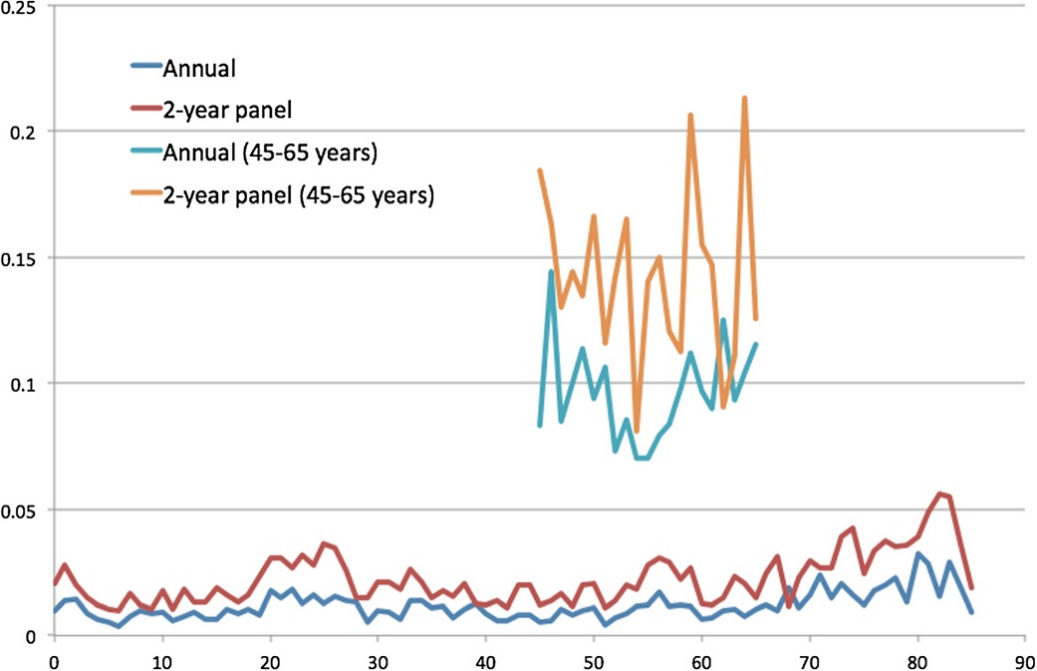
**R-squared estimated from 1- and 2-year emergency room visit prediction by basic demographic characteristics for all ages and by basic and other variables for age 45 to 65 years.**

**Figure 9:**
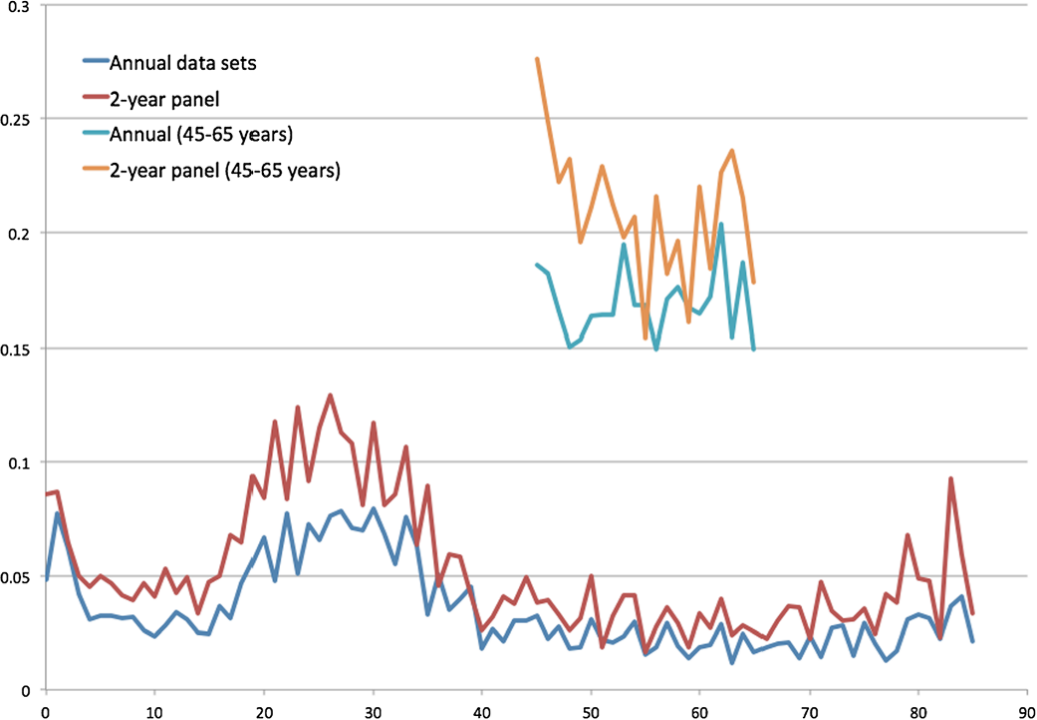
**R-squared estimated from 1- and 2-year office-based visit prediction by basic demographic characteristics for all ages and by basic and other variables for age 45 to 65 years.**

In Figure 2, the ratios of mean spending and standard errors (SEs) were plotted against ages. The ratios of mean spending seem to align with the horizontal line of two, except for lower at age zero, suggesting the spending sum at age zero and one was less than twice the amount at age zero. Most of the SE ratios were above two, except for certain ages. The mean ratios of mean spending and SEs were 2.09 and 2.58.Figure 3 presents the mean emergency room (ER) visits by age. The distribution is not similar to that of health spending. The ER visits were more at age 1 and around age 20. After age 30, ER visits decreased until age 60 to 70. In Figure 4, the ratios of mean visits and SEs were plotted against ages. The mean ratios of mean ER visits and SEs 2.02 and 2.18.Figure 5 presents the mean office-based visits by age and the J-shape distribution is similar to that of health spending. In Figure 6, the ratios of means and SEs were mostly greater than two. The mean ratios of mean office-based visits and SEs were 1.99 and 2.42.Figure 7 presents the estimated R-squared predicted by basic demographic information (gender, race and regions) of all ages and those predicted by basic and other variables from age 45 to 65 years. Because the R-squared from 2-year spending of all ages seemed to be higher of most age groups, the health spending observed in two years might be more predictable than that observed in one year. Figures 8 and 9 present the R-squared predicted from ER and office-based visits. In general, the R-squared was larger if predicted with more variables. The predictability (R-squared) of health spending and office-based visits seemed to be higher at age 20 to 30 years.

## Discussion

### Length of observation and spending distribution

The main findings in this study include (1) spending variation is associated with the lengths of observation time (one- versus two-year observation time); (2) the ratios of mean spending distribution observed, roughly two, in one- and two-year periods seem to match the simplified example assuming normal distributions; and (3) the predictability (R-squared) of total health spending captured in two-year spending models seems to be better than those in one-year models (Figures 7, 8 and 9). However, it is not clear whether the observation time could be a major factor in the predictability (R-squared changes) and ratios of variances of health expenditures in two- and one-year models.

The first strength of this study is that the one- and two-year data used in this study is nationally representative and have comparable quality. The subsequent analysis with mean spending and variances should be representative for the US population from 1996 to 2008. Second, the data collected on all age groups helped to understand the changes of health spending in different stages of life. The shapes of the spending curves by age groups showed properties of health spending: higher spending and variation at older ages and more predictable spending patterns among the elderly (in terms of R-squared) [1]. However, better predictability of office-based visits (R-squared) [1] could not be fully supported if compared to the derived R-squared of health spending and emergency room visits.

On the other hand, there were limitations for the analysis. First, we are not sure about to what extent the example can be applied to the results from MEPS data sets. The real-world data is more complex than this example. The health expenditures incurred in the MEPS data sets included spending due to all health conditions, such as emergency room and office-based visits, and this example only provided a uniform distribution of spending. Then, the link between the example and results from the MEPS data sets was not clear, even when the ratios of mean spending between two- and one-year spending were similar to the predicted values (two) in the most simplistic example (spending observed in two years exactly twice as much as that observed in one year). It is unclear whether higher variance ratios in some age groups suggested influence from other factors.

In conclusion, this study is a first attempt to address possible bias due to lengths of observation time and possible theoretical explanation with a mathematical model using a combination of normal distributions.

## Endnote

^a^The regional variables used in Buntin and Zaslavsky [12] were “counties” where individuals resided. The regional variables in MEPS were “regions” in the US. Because of the differences in how geographic locations were defined and the datasets used in different studies, the AAPCC estimates in this dissertation might not be fully comparable to other studies.

## Electronic supplementary material

Additional file 1: Table S1: Mean health expenditure, emergency room (ER) visits and office-based visits by age groups, estimated from cross-sectional and 2-year panel datasets of the Medical Expenditure Panel Survey from 1996 to 2008. (DOC 192 KB)
