# Supplementary material for: Risk adjustment and observation time: comparison between cross-sectional and 2-year panel data from the Medical Expenditure Panel Survey (MEPS)
Source: Health Inf Sci Syst. 2014 Jul 25;2:5. doi: 10.1186/2047-2501-2-5 (PMC4340859; doi:10.1186/2047-2501-2-5)
Supplement: Supplementary file 1 — Additional file 1: Table S1: Mean health expenditure, emergency room (ER) visits and office-based visits by age groups, estimated from cross-sectional and 2-year panel datasets of the Medical Expenditure Panel Survey from 1996 to 2008. (DOC 192 KB) [file 13755_2013_16_MOESM1_ESM.doc]

Additional file 1: Table S1. Mean health expenditure, emergency room (ER) visits and office-based visits by age groups, estimated from cross-sectional and 2-year panel datasets of the Medical Expenditure Panel Survey from 1996 to 2008.

|  | **Observed in 12 months** | | | | |  | **Observed in 2 years** | | | | | |  |
| --- | --- | --- | --- | --- | --- | --- | --- | --- | --- | --- | --- | --- | --- |
|  | Total health expenditure ($) | | ER visits |  | Office-based visits | | Total health expenditure ($) | | ER visits |  | Office-base visits | |  |
| **Age (years)** | Mean | SE | Mean | SE | Mean | SE | Mean | SE | Mean | SE | Mean | SE | Age (years) |
| **0** | 2,525.08 | 205.26 | 0.13 | 0.01 | 3.59 | 0.07 | 3,514.51 | 263.45 | 0.43 | 0.02 | 8.46 | 0.15 | 0 |
| **1** | 1,433.80 | 110.72 | 0.31 | 0.01 | 4.87 | 0.08 | 2,262.55 | 154.10 | 0.57 | 0.02 | 7.61 | 0.15 | 1 |
| **2** | 902.72 | 114.07 | 0.28 | 0.01 | 3.01 | 0.06 | 1,894.87 | 364.07 | 0.52 | 0.02 | 5.36 | 0.13 | 2 |
| **3** | 828.08 | 78.22 | 0.21 | 0.01 | 2.36 | 0.05 | 1,564.37 | 133.69 | 0.38 | 0.02 | 4.55 | 0.11 | 3 |
| **4** | 678.93 | 37.68 | 0.18 | 0.01 | 2.13 | 0.05 | 1,619.08 | 227.28 | 0.34 | 0.02 | 4.28 | 0.12 | 4 |
| **5** | 737.87 | 39.32 | 0.15 | 0.01 | 2.17 | 0.05 | 1,527.64 | 113.08 | 0.29 | 0.02 | 4.01 | 0.10 | 5 |
| **6** | 715.34 | 37.35 | 0.14 | 0.01 | 1.90 | 0.04 | 1,532.61 | 123.96 | 0.27 | 0.01 | 3.62 | 0.11 | 6 |
| **7** | 841.19 | 67.32 | 0.13 | 0.01 | 1.82 | 0.05 | 1,839.09 | 185.28 | 0.26 | 0.01 | 3.58 | 0.13 | 7 |
| **8** | 749.07 | 39.12 | 0.13 | 0.01 | 1.69 | 0.05 | 1,495.20 | 80.23 | 0.26 | 0.01 | 3.27 | 0.10 | 8 |
| **9** | 863.64 | 89.75 | 0.12 | 0.01 | 1.64 | 0.05 | 1,892.05 | 203.41 | 0.25 | 0.01 | 3.39 | 0.15 | 9 |
| **10** | 909.05 | 58.78 | 0.12 | 0.01 | 1.68 | 0.04 | 1,966.52 | 158.88 | 0.25 | 0.01 | 3.29 | 0.09 | 10 |
| **11** | 982.43 | 64.20 | 0.13 | 0.01 | 1.70 | 0.05 | 2,262.40 | 156.30 | 0.24 | 0.01 | 3.74 | 0.16 | 11 |
| **12** | 1,151.30 | 65.48 | 0.12 | 0.01 | 1.76 | 0.06 | 2,169.68 | 98.04 | 0.26 | 0.02 | 3.33 | 0.10 | 12 |
| **13** | 1,209.71 | 50.18 | 0.13 | 0.01 | 1.69 | 0.05 | 2,523.70 | 131.96 | 0.25 | 0.01 | 3.39 | 0.12 | 13 |
| **14** | 1,241.81 | 63.34 | 0.13 | 0.01 | 1.78 | 0.06 | 2,508.94 | 137.46 | 0.26 | 0.01 | 3.53 | 0.13 | 14 |
| **15** | 1,286.45 | 66.57 | 0.13 | 0.01 | 1.85 | 0.06 | 2,713.39 | 204.17 | 0.27 | 0.01 | 3.74 | 0.13 | 15 |
| **16** | 1,308.76 | 73.09 | 0.16 | 0.01 | 1.91 | 0.06 | 2,649.72 | 127.72 | 0.32 | 0.02 | 3.95 | 0.14 | 16 |
| **17** | 1,202.83 | 48.83 | 0.15 | 0.01 | 1.83 | 0.05 | 2,396.27 | 115.50 | 0.33 | 0.02 | 3.56 | 0.15 | 17 |
| **18** | 1,204.30 | 65.13 | 0.19 | 0.01 | 1.80 | 0.07 | 2,661.63 | 285.42 | 0.41 | 0.02 | 3.50 | 0.14 | 18 |
| **19** | 1,178.66 | 100.14 | 0.20 | 0.01 | 1.72 | 0.06 | 2,437.54 | 149.72 | 0.41 | 0.02 | 3.26 | 0.13 | 19 |
| **20** | 1,391.76 | 228.18 | 0.21 | 0.01 | 1.61 | 0.05 | 2,713.72 | 304.43 | 0.39 | 0.02 | 3.29 | 0.14 | 20 |
| **21** | 1,215.96 | 68.08 | 0.21 | 0.01 | 1.67 | 0.06 | 2,424.25 | 155.08 | 0.42 | 0.03 | 3.19 | 0.14 | 21 |
| **22** | 1,209.68 | 58.80 | 0.21 | 0.01 | 1.66 | 0.06 | 2,573.47 | 134.54 | 0.43 | 0.02 | 3.55 | 0.14 | 22 |
| **23** | 1,209.37 | 57.69 | 0.20 | 0.01 | 1.73 | 0.06 | 2,602.07 | 158.09 | 0.42 | 0.02 | 3.40 | 0.13 | 23 |
| **24** | 1,277.41 | 64.35 | 0.20 | 0.01 | 1.89 | 0.07 | 3,134.41 | 258.74 | 0.38 | 0.02 | 3.93 | 0.17 | 24 |
| **25** | 1,456.97 | 120.11 | 0.19 | 0.01 | 1.91 | 0.07 | 2,924.99 | 162.13 | 0.38 | 0.03 | 3.98 | 0.17 | 25 |
| **26** | 1,414.17 | 67.25 | 0.19 | 0.01 | 2.05 | 0.07 | 2,884.46 | 148.88 | 0.40 | 0.03 | 4.07 | 0.17 | 26 |
| **27** | 1,485.11 | 69.54 | 0.20 | 0.01 | 2.21 | 0.08 | 3,175.54 | 151.85 | 0.37 | 0.02 | 4.62 | 0.20 | 27 |
| **28** | 1,703.98 | 96.14 | 0.20 | 0.01 | 2.34 | 0.08 | 3,690.96 | 274.57 | 0.38 | 0.02 | 4.70 | 0.19 | 28 |
| **29** | 1,730.27 | 97.95 | 0.16 | 0.01 | 2.45 | 0.08 | 3,106.49 | 152.97 | 0.31 | 0.02 | 4.85 | 0.21 | 29 |
| **30** | 1,547.03 | 60.10 | 0.17 | 0.01 | 2.42 | 0.08 | 3,387.38 | 151.37 | 0.35 | 0.02 | 4.86 | 0.19 | 30 |
| **31** | 1,829.30 | 82.46 | 0.18 | 0.01 | 2.56 | 0.08 | 3,747.15 | 270.04 | 0.32 | 0.02 | 5.30 | 0.22 | 31 |
| **32** | 1,990.06 | 126.59 | 0.16 | 0.01 | 2.69 | 0.08 | 4,290.23 | 331.78 | 0.32 | 0.02 | 5.44 | 0.21 | 32 |
| **33** | 2,077.57 | 121.53 | 0.17 | 0.01 | 2.75 | 0.08 | 4,099.57 | 196.30 | 0.33 | 0.02 | 5.27 | 0.20 | 33 |
| **34** | 1,832.69 | 70.59 | 0.15 | 0.01 | 2.65 | 0.09 | 3,547.54 | 155.32 | 0.30 | 0.02 | 5.22 | 0.21 | 34 |
| **35** | 1,692.08 | 63.53 | 0.17 | 0.01 | 2.55 | 0.09 | 3,476.75 | 151.61 | 0.33 | 0.02 | 4.89 | 0.16 | 35 |
| **36** | 1,844.32 | 78.07 | 0.17 | 0.01 | 2.55 | 0.08 | 4,013.86 | 202.48 | 0.36 | 0.02 | 5.33 | 0.21 | 36 |
| **37** | 2,156.19 | 113.56 | 0.17 | 0.01 | 2.73 | 0.08 | 4,352.14 | 272.61 | 0.31 | 0.02 | 5.38 | 0.23 | 37 |
| **38** | 2,002.03 | 92.13 | 0.15 | 0.01 | 2.69 | 0.09 | 4,217.82 | 264.77 | 0.31 | 0.02 | 5.26 | 0.19 | 38 |
| **39** | 2,112.27 | 128.89 | 0.17 | 0.01 | 2.75 | 0.08 | 4,763.66 | 779.99 | 0.32 | 0.02 | 5.61 | 0.24 | 39 |
| **40** | 2,068.16 | 87.88 | 0.15 | 0.01 | 2.88 | 0.11 | 4,498.81 | 271.82 | 0.30 | 0.02 | 5.60 | 0.25 | 40 |
| **41** | 2,223.92 | 114.79 | 0.16 | 0.01 | 2.95 | 0.09 | 4,821.78 | 284.98 | 0.33 | 0.02 | 5.72 | 0.22 | 41 |
| **42** | 2,389.40 | 184.71 | 0.16 | 0.01 | 2.77 | 0.09 | 5,302.66 | 967.05 | 0.30 | 0.02 | 5.61 | 0.21 | 42 |
| **43** | 2,549.81 | 231.47 | 0.15 | 0.01 | 2.90 | 0.08 | 4,946.25 | 283.24 | 0.30 | 0.02 | 5.76 | 0.19 | 43 |
| **44** | 2,224.88 | 86.90 | 0.14 | 0.01 | 2.83 | 0.08 | 4,845.03 | 229.33 | 0.28 | 0.02 | 5.74 | 0.21 | 44 |
| **45** | 2,525.85 | 130.60 | 0.15 | 0.01 | 2.96 | 0.08 | 5,396.13 | 421.42 | 0.32 | 0.02 | 5.98 | 0.19 | 45 |
| **46** | 2,584.06 | 104.75 | 0.16 | 0.01 | 3.16 | 0.09 | 5,568.95 | 249.63 | 0.33 | 0.02 | 6.38 | 0.22 | 46 |
| **47** | 2,771.29 | 103.10 | 0.16 | 0.01 | 3.32 | 0.10 | 6,112.62 | 300.46 | 0.35 | 0.02 | 6.85 | 0.23 | 47 |
| **48** | 3,013.89 | 121.78 | 0.18 | 0.01 | 3.59 | 0.11 | 6,294.30 | 336.06 | 0.33 | 0.02 | 7.07 | 0.27 | 48 |
| **49** | 3,080.24 | 121.41 | 0.14 | 0.01 | 3.49 | 0.09 | 6,511.14 | 289.81 | 0.31 | 0.02 | 7.05 | 0.24 | 49 |
| **50** | 3,065.79 | 110.15 | 0.17 | 0.01 | 3.55 | 0.09 | 6,522.97 | 325.12 | 0.33 | 0.02 | 7.20 | 0.23 | 50 |
| **51** | 3,491.52 | 162.53 | 0.15 | 0.01 | 3.88 | 0.12 | 7,594.47 | 398.27 | 0.31 | 0.02 | 7.92 | 0.28 | 51 |
| **52** | 3,476.07 | 120.97 | 0.16 | 0.01 | 3.91 | 0.09 | 7,626.17 | 424.70 | 0.31 | 0.02 | 7.51 | 0.24 | 52 |
| **53** | 3,749.43 | 229.26 | 0.16 | 0.01 | 3.91 | 0.10 | 7,599.51 | 341.70 | 0.33 | 0.02 | 7.98 | 0.27 | 53 |
| **54** | 3,871.51 | 154.97 | 0.17 | 0.01 | 4.04 | 0.10 | 8,322.34 | 429.23 | 0.35 | 0.02 | 8.16 | 0.24 | 54 |
| **55** | 4,423.06 | 219.30 | 0.16 | 0.01 | 4.29 | 0.12 | 8,749.36 | 397.99 | 0.31 | 0.02 | 8.64 | 0.30 | 55 |
| **56** | 4,450.83 | 175.43 | 0.17 | 0.01 | 4.28 | 0.11 | 9,958.75 | 535.12 | 0.33 | 0.02 | 8.82 | 0.30 | 56 |
| **57** | 4,545.50 | 219.62 | 0.16 | 0.01 | 4.24 | 0.11 | 10,061.53 | 565.49 | 0.31 | 0.02 | 8.54 | 0.29 | 57 |
| **58** | 4,990.20 | 240.64 | 0.16 | 0.01 | 4.65 | 0.14 | 10,773.29 | 793.18 | 0.36 | 0.03 | 9.49 | 0.34 | 58 |
| **59** | 5,472.13 | 491.82 | 0.20 | 0.01 | 4.87 | 0.14 | 10,888.13 | 1,349.84 | 0.34 | 0.02 | 9.56 | 0.38 | 59 |
| **60** | 4,785.10 | 337.80 | 0.17 | 0.01 | 4.84 | 0.14 | 10,160.97 | 492.72 | 0.38 | 0.03 | 9.91 | 0.36 | 60 |
| **61** | 4,988.29 | 227.34 | 0.17 | 0.01 | 4.83 | 0.13 | 10,151.25 | 559.97 | 0.31 | 0.02 | 9.57 | 0.32 | 61 |
| **62** | 5,326.18 | 316.90 | 0.17 | 0.01 | 4.96 | 0.15 | 12,714.64 | 895.66 | 0.35 | 0.02 | 10.51 | 0.35 | 62 |
| **63** | 6,121.18 | 311.59 | 0.18 | 0.01 | 5.43 | 0.16 | 11,197.49 | 580.31 | 0.35 | 0.02 | 10.66 | 0.40 | 63 |
| **64** | 5,694.42 | 265.34 | 0.17 | 0.01 | 5.43 | 0.15 | 12,181.57 | 620.82 | 0.36 | 0.03 | 11.00 | 0.42 | 64 |
| **65** | 5,821.72 | 261.30 | 0.19 | 0.01 | 5.50 | 0.17 | 11,693.01 | 589.08 | 0.38 | 0.03 | 10.91 | 0.35 | 65 |
| **66** | 5,882.83 | 257.86 | 0.19 | 0.01 | 5.97 | 0.19 | 12,377.96 | 608.90 | 0.40 | 0.02 | 12.05 | 0.40 | 66 |
| **67** | 5,912.41 | 266.08 | 0.21 | 0.02 | 6.17 | 0.20 | 11,614.87 | 619.61 | 0.41 | 0.04 | 12.08 | 0.44 | 67 |
| **68** | 5,709.24 | 282.52 | 0.19 | 0.01 | 6.22 | 0.18 | 12,097.51 | 666.53 | 0.35 | 0.03 | 12.41 | 0.46 | 68 |
| **69** | 5,673.66 | 255.65 | 0.19 | 0.02 | 6.30 | 0.18 | 12,159.49 | 611.85 | 0.42 | 0.03 | 12.64 | 0.52 | 69 |
| **70** | 6,098.07 | 255.85 | 0.20 | 0.01 | 6.24 | 0.17 | 13,956.91 | 840.27 | 0.44 | 0.03 | 13.11 | 0.49 | 70 |
| **71** | 6,946.53 | 373.33 | 0.24 | 0.02 | 7.01 | 0.20 | 14,788.19 | 851.20 | 0.50 | 0.04 | 14.39 | 0.48 | 71 |
| **72** | 7,027.09 | 334.22 | 0.21 | 0.02 | 6.82 | 0.18 | 14,257.72 | 730.21 | 0.38 | 0.03 | 14.05 | 0.47 | 72 |
| **73** | 6,958.63 | 364.37 | 0.20 | 0.02 | 7.13 | 0.22 | 13,594.54 | 604.74 | 0.45 | 0.03 | 13.98 | 0.44 | 73 |
| **74** | 6,792.98 | 327.94 | 0.22 | 0.02 | 7.25 | 0.20 | 13,942.20 | 636.28 | 0.48 | 0.03 | 14.41 | 0.51 | 74 |
| **75** | 7,593.97 | 468.09 | 0.24 | 0.02 | 7.37 | 0.20 | 15,248.39 | 862.57 | 0.46 | 0.03 | 14.93 | 0.47 | 75 |
| **76** | 7,645.93 | 400.09 | 0.26 | 0.02 | 7.67 | 0.28 | 15,435.06 | 887.15 | 0.50 | 0.04 | 15.29 | 0.65 | 76 |
| **77** | 6,789.98 | 295.77 | 0.27 | 0.02 | 7.88 | 0.26 | 14,161.83 | 652.08 | 0.56 | 0.04 | 15.84 | 0.57 | 77 |
| **78** | 7,728.76 | 396.93 | 0.28 | 0.02 | 7.92 | 0.22 | 17,256.88 | 1,009.82 | 0.62 | 0.05 | 14.90 | 0.55 | 78 |
| **79** | 7,851.32 | 345.60 | 0.31 | 0.03 | 7.54 | 0.24 | 17,636.82 | 1,781.89 | 0.56 | 0.04 | 15.06 | 0.54 | 79 |
| **80** | 8,304.24 | 394.91 | 0.30 | 0.02 | 7.89 | 0.22 | 15,966.14 | 830.02 | 0.55 | 0.05 | 15.85 | 0.54 | 80 |
| **81** | 7,593.81 | 415.21 | 0.31 | 0.03 | 7.83 | 0.24 | 17,254.65 | 1,051.26 | 0.67 | 0.07 | 15.02 | 0.56 | 81 |
| **82** | 9,031.23 | 471.52 | 0.31 | 0.02 | 7.77 | 0.27 | 18,056.52 | 1,176.34 | 0.62 | 0.05 | 14.66 | 0.57 | 82 |
| **83** | 7,987.46 | 437.28 | 0.33 | 0.03 | 7.36 | 0.23 | 17,407.37 | 1,483.12 | 0.68 | 0.07 | 15.02 | 0.66 | 83 |
| **84** | 8,003.69 | 384.12 | 0.33 | 0.03 | 7.48 | 0.28 | 16,660.60 | 1,050.51 | 0.64 | 0.04 | 13.92 | 0.71 | 84 |
| **85** | 9,184.51 | 262.22 | 0.40 | 0.02 | 7.19 | 0.19 | 18,782.21 | 677.04 | 0.83 | 0.04 | 13.92 | 0.44 | 85 |
